# Editorial: Crustacean reproductive physiology and its applications

**DOI:** 10.3389/fphys.2022.1018481

**Published:** 2022-09-15

**Authors:** Haihui Ye, Chaoshu Zeng, Naoaki Tsutsui, Heinrich Dircksen

**Affiliations:** ^1^ College of Fisheries, Jimei University, Xiamen, China; ^2^ College of Science and Engineering, James Cook University, Townsville, QLD, Australia; ^3^ Department of Marine Bioresources, Faculty of Bioresources, Mie University, Tsu, Japan; ^4^ Department of Zoology, Stockholm University, Stockholm, Sweden

**Keywords:** reproduction, metabolic regulation, growth, genetics, neuropeptides, crustacea

Crustacea constitute an important taxonomic group in aquatic ecosystems and form an important sector of aquaculture industry. Novelties in studies on crustacean reproductive physiology help shedding new lights on deeper understanding of the mechanisms of sex determination and differentiation of crustaceans. The application of established and innovative techniques based on such knowledge will contribute significantly to progress in the crustacean aquaculture industry. The objective of this Special Issue was to provide a forum for researchers to report upon their cutting-edge research in Crustacean Reproductive Physiology and its Applications. This Research Topic comprises ten original research articles.

In particular, in this Special Issue, the roles of several neuropeptides in regulating crustacean reproduction have been reported for several decapod crustacean species of importance for aquaculture. In the giant freshwater prawn *Macrobrachium rosenbergii*, Ao et al. revealed that recombinant neuroparsins NP1 and NP2 stimulate expression of the vitellogenin (Vg) gene, and silencing of NP1 and NP2 genes suppresses Vg, Vg receptor, and CyclinB gene expressions. In the mud crab *Scylla olivacea*, double strand RNA technology (dsRNA) was used to inhibit transcription of vitellogenesis-inhibiting hormone (VIH), i.e. dsRNA-VIH accelerates ovarian maturation by increasing hemolymph vitellogenin concentration and the gonadosomatic index (Duangprom et al.). In the swimming crab *Portunus trituberculatus*, Tu et al. confirmed via *in vitro* experiments that the expressions of Vg, VgR, cyclinB, and Cdc2 in ovary explants is induced by synthetic corazonin, but reduced by corazonin receptor dsRNA. Tu et al. also suggested that the corazonin/corazonin receptor signaling system stimulates the biosynthesis of ecdysteroids. It is known that crustacean female sex hormone (CFSH) plays a pivotal role in the development of secondary sex characteristics in dioecious species. The roles of CFSH were reported for the first time in female reproductive physiology in the hermaphrodite cleaner shrimp *Lysmata vittata* in this Special Issue. CFSH1a was found to be indispensable for the development of female gonopores, but was probably not involved in the control of vitellogenesis in this species. In terms of male reproductive physiology, CFSH1a appeared to suppress the mRNA expression of the insulin-like androgenic gland hormone 2 (IAG2) in short-term silencing and recombinant protein injection experiments. However, CFSH1a did not affect male sexual differentiation in long-term silencing experiments (Liu et al.).

Up to the present date, autocrine and paracrine mechanisms are poorly understood in crustaceans. Thus, as an important novelty in this Special Issue, Yang et al. revealed that in the ovary of the mud crab *Scylla paramamosain*, bone morphogenetic protein 2 (BMP2) is exclusively detected in oocytes, whereas BMP2-receptors are expressed in both follicle cells and oocytes. RNAi tests further suggested that BMP2 promotes oocyte maturation through an autocrine/paracrine pathway. It is reasonable but novel that autocrine/paracrine regulation of gonadal function by the transforming growth factor β (TGFβ) superfamily, which is well known in vertebrates, also exists in crustaceans.

RNA -Seq is a powerful tool for uncovering molecular events in gonadal development. Wang et al. applied full-length transcriptome sequencing and comparative transcriptomic analysis to provide insights into the ovarian maturation of the ridge tail white shrimp *Exopalaemon carinicauda*. Li et al. identified a novel Vg specifically expressed in the hepatopancreas of the Pacific white shrimp *Litopenaeus vannamei*, and revealed an exogenous nutrient transfer and accumulation mechanism from the hepatopancreas to ovary in Penaeid shrimps.

Male reproductive physiology in crustaceans often appears to attract too little attention. In this Special Issue, RNAi analyses in the oriental river prawn *Macrobrachium nipponense* revealed that ferritin positively affects mRNA expression of IAG and the secretion of testosterone, thus positively affecting testis development in this species (Jin et al.).

In addition, the genetic mechanism of sex determination in the Chinese shrimp *Fenneropenaeus chinensis* was explored by Wang et al. The authors applied resequencing of data to detect sex-linked variants and female-specific sequences, and found these clearly suggestive of a female heterogametic (ZW) sex determination system.

Copepods are small planktonic crustaceans often serving as excellent prey for larval rearing in hatcheries. Wang et al. investigated the embryonic development and effects of temperature, salinity and light intensity on egg hatching of the calanoid copepod *Bestiolina amoyensis*, and suggested that *B. amoyensis* is a good candidate as live feed for larval rearing.

We hope the readers will benefit from these articles in their research and enjoy reading these subjects as much as we did while editing them. We sincerely thank all authors and reviewers for their participation and commitment that made publication of this Research Topic possible.

